# Teeth in Diabetes

**Published:** 1891-09

**Authors:** 


### Teeth in Diabetes.

The importance of early diagnosis in diabetes can not be overestimated because the prognosis may be much influenced by the institution of early treatment. It is interesting, therefore, to call attention to the fact that the *Wiener Medizinische Presse*, January 4, 1891, contains a brief abstract of a publication by Magitot in *Bulletin Medical*, in which he dwells upon the diagnostic significance of alveolar osteo-periostitis as a manifestation of diabetes. He has noticed in this disease a deviation of the tooth which later becomes loosened. At the same time an alveolar catarrh exists.

Ultimately the tooth falls out. In the further course of the disease the alveolar border of the bone may be absorbed, and this in turn may be followed by gangrene of the gum.—*Medical and Surgical Reporter*.

